# Investigating the Effect of School Bus Stopping Process on Driver Behavior of Surrounding Vehicles Based on a Driving Simulator Experiment

**DOI:** 10.3390/ijerph182312538

**Published:** 2021-11-28

**Authors:** Yanyan Chen, Yinjia Guo, Xin Gu, Yuntong Zhou, Yao Tong, Bingxin Cao

**Affiliations:** Beijing Key Laboratory of Traffic Engineering, Beijing University of Technology, Beijing 100124, China; cdyan@bjut.edu.cn (Y.C.); guoyinjia@emails.bjut.edu.cn (Y.G.); yuntong@emails.bjut.edu.cn (Y.Z.); tongyao19980930@163.com (Y.T.); caobingxin@emails.bjut.edu.cn (B.C.)

**Keywords:** school bus, driving simulator, driving behavior, school bus regulations

## Abstract

School bus safety has attracted widespread attention with economic development and the improvement of overall quality of the population. However, violations of school bus regulations and school bus-related crashes often occur. Limited research has been conducted on the impact of the school bus stopping process on surrounding drivers’ behavior. This study provides a driving simulator experiment to explore drivers’ behaviors during the school bus stopping process under different traffic law awareness status, traffic volume status, and initial location status. Eight variables about behavior decision and kinetic parameters were assessed for analysis by a logistic regression model and multivariate analysis of variance (MANOVA). Results show that the mean speed decreases and the number of people complying with the regulations increases after publicizing the regulations. The proportion of surrounding vehicles in the acceleration state increases, especially under the scenario that the traffic volume is large and the initial distance is far. This indicates that the enforcement of the regulations may stimulate unsafe driving behavior. The findings of this study could help policy makers to better understand the prevalence and compliance of current school bus stopping regulations among drivers and support improvements in the practical application of the regulations.

## 1. Introduction

School buses have long been a popular way for students to commute to school in various countries. According to statistics, there are 450,000 school buses in operation in the United States [1]. Recently, with the emphasis on child safety and the popularization of school buses in developing countries, the number of school buses and the annual growth rate are increasing year by year [2,3]. In China, data show that the number of school buses has reached 164,635, serving a total of 93,495,552 students in both compulsory and preschool education. However, the popularity of school buses also brings the corresponding safety problems. According to statistics from the United States, approximately 140 school-age children die and 85,000 are injured each year due to school bus-related crashes [4], and a decade of data indicate that the increase in vehicle safety systems has not contributed to a reduction in fatal school bus-related crashes [5]. In China, according to statistics from the school bus website, 339 school bus safety violations were reported between 2012–2017, resulting in 801 casualties, including 156 deaths [6]. Therefore, it is necessary to conduct in-depth analysis of school bus-related crashes and take measures to improve school bus safety.

According to the literature, the causes of school bus-related crashes are mainly classified into three categories: human factors, vehicle factors, and road factors [3]. Researchers have further divided school bus-related crashes into “active crashes” and “passive crashes” due to different reasons [7]. The occurrence of active crashes is mainly related to human factors and vehicle factors of school bus itself, such as the lack of safety awareness of the drivers and passengers, as well as the safety of the school bus itself and the legality of operation. The occurrence of passive crashes is related to poor road conditions and other traffic environment factors such as non-compliance of school bus privileges by other vehicles [3]. Numerous previous studies have been conducted to improve the safety of school buses; however, most of them have focused on studying measures related to school bus itself and school bus management. For example, some scholars focused on the design of school buses and safety [7,8,9]; some scholars focused on how to use intelligent transportation systems to monitor school buses and provide assistant strategies [2,10,11]; others conducted research on the development of school bus management systems [12]. On the whole, the above research on school bus safety has played a significant role in ensuring the safety of passengers in school buses. Evidence has shown that school bus occupants are at a lower risk of fatal injuries compared to other people involved in school bus-related crashes [13,14,15,16].

However, school bus crashes caused by surrounding drivers have not been resolved, and the consequences of these crashes are serious [3]. Results from previous research show that the occurrence of such crashes is mostly related to the fact that the drivers around the school bus do not understand or comply with the “school bus privileges”, thus illegally passing the stopped school bus [4], as well as failing to maintain a safe following distance [3]. These illegal behaviors of surrounding vehicles tend to result in traffic crashes, such as collisions with children getting off the school bus and trying to cross the road, and sideswipe or rear-end collisions with the school bus or other vehicles around the school bus. A field study conducted in Florida confirmed that illegal overtaking by other drivers is the biggest safety problem faced by school buses [1]. Hence, further understanding of the behavior of surrounding vehicles during the stopping process of the school bus (the process from the beginning of the slowdown to a complete stop) is crucial to improve the safety of the school bus. 

To ensure school bus safety, various countries have introduced appropriate school bus safety regulations and policies. In the U.S., although the details of the rules vary from state to state, generally all vehicles in both directions must stop when a school bus stops, flashes its red lights, or displays the stop sign. These regulations and policies aim to protect children getting on and off the bus, especially in light of the fact that younger children may not know the rules of the road and may wander off. When a school bus starts, turns off the red light, and puts away the stop sign, other vehicles can restart. The state of Virginia specifically requires that surrounding vehicles must stop when the bus stops, regardless of whether the red lights are on and the stop sign is out. Since 2012, China has also promulgated the School Bus Safety Management Regulations, including that when a school bus stops on a road to pick up or drop off students, it should stop on the right side of the road and turn on its hazard warning flashers and its stop signs. When a school bus stops on a road with only one motorway lane in the same direction, the vehicle behind should stop and wait and should not overtake. When a school bus stops on a road with more than two lanes in the same direction, motor vehicles behind the bus in the stopping lane and in the adjacent lane should stop and wait, and motor vehicles in other lanes should slow down to pass. Motor vehicles waiting behind the school bus should not sound their horns or use their lights to hurry the school bus. 

In an ideal state, law on stopping for a school bus issued by various countries can reduce corresponding crashes. However, non-compliance with regulations often occurs. Appachu et al. [4] identified 10,590 cases of vehicles illegally passing 3427 school buses in a single day. Studies have shown that the potential danger posed by violations is a major cause of road crashes [17,18,19]. Additionally, according to data analysis, from 2014, the number of casualty crashes on school buses in China still increased year by year, and the casualty and death rates of single crash increased from 3.5 and 0.75 per crash in 2013 to 13.38 and 2.25 per crash in 2016 [3]. So, are school bus regulations doing what they are supposed to do? This is a question to ponder.

A questionnaire survey was conducted to investigate the awareness of school bus regulations in China, covering people from different provinces. Results from questionnaire survey showed that only 37.8 percent of participants were aware of school bus stopping regulations, and only 16.2 percent of those had complied with school bus regulations in their previous driving experience. In addition, we investigated the situations influencing drivers’ willingness to comply with the regulations and found that three situations would contribute to drivers violate the regulations: (1) when other vehicles around them do not comply with the regulations; (2) when they are on their way to or from work, or have time pressure; (3) when the road is congested due to the school bus. It has been suggested that most of the crashes have been blamed on driver’s errors and violations of the traffic regulations; therefore, complying with acceptable driving norms and regulations can have the mobility effect of improving the overall safety of the road [20].

Therefore, in this study we want to explore three questions: (1) How does the school bus stopping process affect surrounding drivers’ behavior? (2) Will the driver’s traffic law awareness (knowledge of school bus regulations) affect driving behavior? (3) Will different traffic environments affect surrounding drivers’ behavior? To this end, a driving simulator experiment is designed and conducted to study these issues. 

The structure of the remainder of this paper is as follows. Section 2 presents the experiment design and data collection. Section 3 introduces the model estimation and results. The following section discusses the problem. The last section presents the concluding remarks and future research directions.

## 2. Methods

### 2.1. Participants

A total of 41 voluntary application participants were recruited for this study by advertising in school forums and social media. They were required to hold a valid driving license with at least one-year driving experience. Excepting four drivers who did not complete the experiment due to motion sickness, 37 participants were finally involved, including 21 male drivers and 16 female drivers, while they were from different occupations, such as teachers, company employees, workers and students, which makes the experimental group more representative of the entire driving population during the school bus travel period of our study. Subjects of different driving ages were selected, due to the heterogeneity in traffic behavior and the risk of road traffic crashes [21,22,23]. In addition, this study also considered a balanced ratio of males to females in the selection of subjects [21,24,25]. The drivers’ ages ranged from 21 to 46, with an average age of 28.6 years and a median age of 27 years. The number of years since they had obtained the driving license ranged from 2 to 18 years, with an average of 5 years at the time of this experiment. Moreover, informed consent was obtained from each participant before the experiment and the data were collected anonymously. This study was conducted in accordance with Chinese laws regarding scientific research on human volunteers.

### 2.2. Apparatus

**Figure 1:**
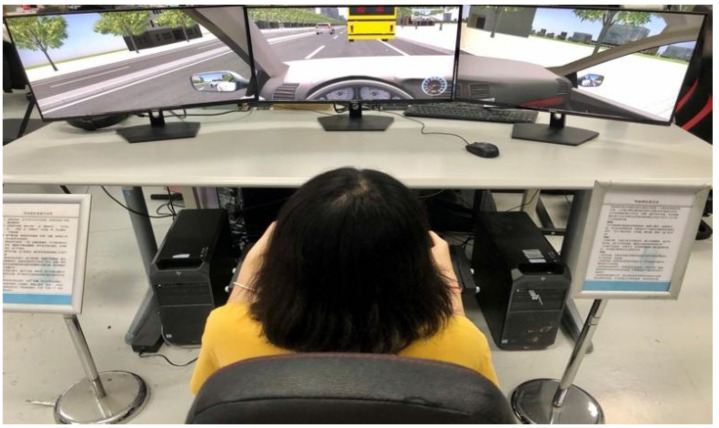
Driving simulator.

In this study, a fixed-base driving simulator at the Key Laboratory of Traffic Engineering of Beijing University of Technology (BJUT) was used to conduct the experiment (Figure 1). The driving simulator provides participants with a 130-degree front field of view as well as a left, middle, and right rear-view mirror through three high-resolution display screens [26,27]. Besides, computer speakers were used to imitate the sounds of vehicles and roads. The participants interacted with the simulator through a set of Logitech steering wheel and pedals, which were calibrated and verified separately before the experiment.

### 2.3. Scenario Design

**Figure 2:**
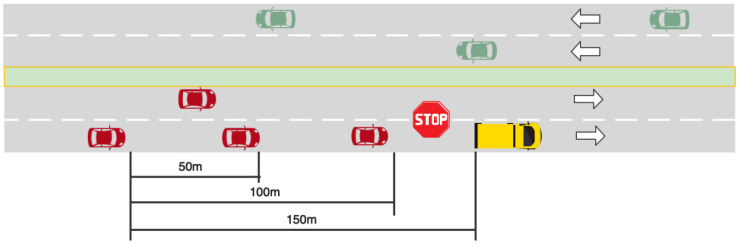
Design of the scenarios.

In this study, driving scenarios were designed to simulate situations in which a school bus stops on the side of the road and to understand how traffic law awareness and the traffic operation environment affect driving behaviors. According to a field survey and literature research, school buses usually stop on a road with only one or two motorway lanes in the same direction and there are few school bus stop signs on the roadside. Thus, the experimental scenario was simulated as a typical urban road with only two motorway lanes in the same direction, each lane being 3.5 m wide and with a speed limit of 70 km/h, separated by a central barrier, as shown in Figure 2. To provide a more realistic environment, the scenario included modeling of the environment around the road appurtenances and simulation of the traffic situation in the opposite carriageway.

Additionally, the experiment was designed as a 2 × 3 × 3 mixed factorial design with traffic law awareness (knowing the law, not knowing the law) as a within-subject variable, and the initial distance from the test car to the school bus (50, 100 and 150 m) as well as traffic volume (500, 1000, and 1500 pcu/h) as between-subject variables. Therefore, the treatments of this experiment are the initial distance from the test car to the school bus (IDCTS) with three different settings (50, 100, and 150 m), and the traffic volume with three different settings (500, 1000, and 1500 pcu/h). Since traffic volume affects the driving behavior of drivers, and as traffic volume increases, the increased interaction between vehicles leads to higher crash risk for drivers [28,29]. Therefore, traffic volume was selected as one of the environmental variables in the study. Besides, in order to investigate the effect of different reactions and the timing of the choice of driving behaviors, different initial distances from the school bus were selected as another environmental variable in the study. The selection of IDCTS parameter values mainly takes into account the driver’s safe following distance and safe lane-changing distance, while the setting of traffic volume parameter values takes into account the road traffic volume corresponding to the different departure times of school buses. 

Each participant was required to individually test each of the combinations of the two treatments separately before and after learning about the school bus regulations. The experiment consisted of nine scenes in total, which were switched using a predetermined experiment script. In this experiment, three identical roads were built for setting three different traffic volumes. On each road above, three experiments with different initial distances from the school bus were completed. At the beginning of each scenario, the test driver’s vehicle was generated in the rightmost lane (the same lane as that of the school bus), and at the same time, a traffic flow with an average speed of 60 km/h was generated. The school bus would appear in front of the test vehicles and would run at a speed of 50 km/h (Chinese regulations stipulate that school buses traveling on normal urban roads should not exceed 60 km/h). When the test vehicle traveled 200 m, the school bus began to decelerate at a deceleration rate of 1.5 m/s^2^ until its speed reached zero, and the entire deceleration process lasted 65 m. 

In the experimental scenario, there are two behavioral choices for the test vehicles during the whole duration of the school bus stopping process (from the time the school bus starts to slow down to the time it stops). One is to stop and wait behind the school bus and the other is to overtake it. For vehicles choosing to stop, the study duration is between the test vehicle starting the journey and the test vehicle stopping behind the school bus. For vehicles choosing to overtake, the study duration is between the test vehicle starting the journey and the rear of the test vehicle overtaking the front of the school bus.

### 2.4. Procedure

Each participant’s experiment includes three phases: pre-experiment, formal experiment, and post-experiment. During the pre-experiment, each participant was briefly introduced to the requirements of the experiment [30]. Then, they were requested to complete an informed consent form and a background information questionnaire. Besides, each participant was trained for 10–15 min to adapt to the simulator usage. The driver’s ability to control speed comfortably and complete lane change as well as other driving operations smoothly were used as the basis for ending the training and starting the formal experiment. 

In the formal experiment, the participants were advised to drive in the same lane as the school bus. The participants were told to drive as they would drive in real life when encountering a school bus in the process of stopping. After completing the first set of experiments, the drivers were educated about school bus stopping regulations, after which they were told they needed to follow the regulations to complete the second set of nine scenarios. The order of experiments in each set was randomized in order to eliminate learning effects. It took approximately 15–20 min to complete the two set of experiments, with a break of at least 5 min at the end of one set of experiments. The participants could quit the experiment at any time in case of motion sickness.

During the post-experiment, each participant needed to complete a questionnaire that collected the subjective feelings of the experiment, including feedback on the realism of the driving simulator and scenario experience. In addition, the participants who completed the whole experiment were paid 200 RMB (about $30) as compensation.

### 2.5. Dependent Variables

Eight key variables, including three categorical variables and five continuous variables, were defined and extracted to evaluate the participants’ driving behavior during the stopping process of the school bus. These variables are explained as follows:

- Stop/Go decision (Stop = 0; Go = 1): This variable was defined as the driver’s decision of whether to stop or go after the school bus’s brake light comes on. If the driver continues driving after the school bus’s brake light comes on, Stop/Go decision = 1, and 0 otherwise.
- Acc/Dec state (Dec = 0; Acc = 1): This variable was defined as the overtaking state the drivers would take when approaching the school bus. For drivers who decide to continue going after the school bus’s brake light comes on, if the driver is accelerating when passing the school bus, the value of this variable is 1.
- Stop/Go bus state (Stop = 0; Go = 1): This variable was defined as the state of the school bus at the moment the vehicle passed the bus. Stop/Go bus state = 0 if the school bus is completely stationary while the test vehicle is passing the school bus.
- Mean speed: The average speed of the test vehicles within the whole duration of school bus stopping process (m/s).
- Max Long Acc: The maximum value of the longitudinal acceleration of the vehicle while traveling behind the school bus (m/s^2^).
- Max Long Dec: The maximum value of the longitudinal deceleration of the vehicle while traveling behind the school bus (m/s^2^).
- Max Lateral Acc: The maximum lateral acceleration of the vehicle while traveling behind the school bus (m/s^2^).
- Percentage of Following Times: The percentage of time that vehicles travel in the same lane as that of the school bus as a percentage of total time spent driving before overtaking the school bus (%).

### 2.6. Statistical Analysis

Multivariate analysis of variance (MANOVA) was used in this study to investigate the effects of factors such as knowledge of school bus stopping regulations, IDCTS, and traffic volume on drivers’ driving behavior indicators. Besides, logistic regression analysis was applied to examine the frequencies of Stop/Go decisions, Acc/Dec state, and Stop/Go bus state results under different driving scenarios. To ensure more reliable results, non-significant variables were first screened out by *t*-tests, and a 95% significance level was used in this study to compare statistical differences.

## 3. Results

During the formal experiment, 666 (37 subjects × 9 test scenarios × 2 situations) trial were conducted in this study. The basic statistical descriptions of key variables in this study are shown in Table 1 and Table 2. In the subsequent analysis, logistic regression models were used to analyze the relationship between Acc/Dec state and Stop/Go bus state among various factors (Table 3). MANOVA was used to investigate the differences in driving behavior among different factors (Table 4).

### 3.1. Categorical Variables

For each test scenario, drivers need to make Stop/Go decision and accelerate/decelerate decision while the school bus slows down and stops. These decisions can reflect the drivers’ behavioral trends toward school bus stopping. The percentages of drivers’ Stop/Go decision, Acc/Dec state, and Stop/Go bus state under different test scenarios are presented in Table 1.

Overall, before the regulations were introduced, on average, only 1% of drivers chose to stop in each scene. Among drivers who chose to overtake the school bus, 52% were in an Acc state and 42% were in a Dec state while approaching the school bus. When the test vehicle overtook, the school bus had stopped completely in 58% of the cases. However, after the regulations were introduced, 12% of vehicles chose to stop, which is a slight increase. Among drivers who chose to overtake the school bus, 66% of drivers were in an Acc state and 34% were in a Dec state while approaching the school bus. When the test vehicle overtook, the school bus had stopped completely in 63% of the cases. Furthermore, it can be inferred from Table 1 that with the knowledge of the regulations, the percentage of Stop decisions at the same IDCTS increased as the traffic volume increased—for example, when the IDCTS was 150 m, the percentage of Stop decisions at a traffic volume of 1500 pcu/h (27%) was greater than at 500 pcu/h (2.7%). At the same time, the percentage of Stop bus states also shows an increasing trend—e.g., at an IDCTS of 150 m, the percentage of Stop bus states was greater than 500 pcu/h (55.6%) at a traffic volume of 1500 pcu/h (66.7%).

Furthermore, three logistic regression models were established to investigate the significant variables. In general, the regression models were statistically significant for the Stop/Go decision (chi-square = 64.362, *p* < 0.001), Acc/Dec state (chi-square = 175.763, *p* < 0.001), and Stop/Go bus state (chi-square = 175.763, *p* < 0.001). The results of the Hosmer and Lemeshow test showed that the logistic regression models have high goodness-of-fits for the Stop/Go decision (chi-square = 0.0390, *p* = 0.843 > 0.05) and Stop/Go bus state (chi-square = 7.366, *p* = 0.392 > 0.05). Table 3 shows the results of three logistic regression models. 

For Stop/Go decision, the results suggested that both traffic law awareness and IDCTS have significant effects on the decision. The variable “know law” (B = −2.5) had a negative relationship with the Stop/Go decision, and IDCTS also had a negative effect on the Stop/Go decision. For Acc/Dec state, knowing the traffic law increased the probability of drivers accelerating while overtaking the school bus. For Stop/Go bus state, gender, traffic volume, and IDCTS were all found to be significant factors. The variable male and moderate traffic (1000 pcu/h) had a negative relationship with Stop/Go bus state, while other factors (1500 pcu/h and IDCTS) were found to be positively correlated with Stop/Go bus state. These results indicated that high traffic volume and large IDCTS might increase the difficulty for drivers to judge the state of the school bus.

### 3.2. Continuous Variables

#### 3.2.1. Mean Speed

The results of the MANOVA (see Table 4) suggested that the drivers’ mean speed while approaching the school bus before overtaking was significantly different before and after learning about the stop regulations of the school bus (F = 5.887, *p* < 0.05), for different traffic volumes (F = 8.179, *p* < 0.001), and for different IDCTS (F = 5.868, *p* < 0.05). Besides, the drivers’ mean speed after learning about the regulations (M = 13.686 m/s, S.D. = 2.42 m/s) was lower than the mean speed before learning about the regulations (M = 14.112 m/s, S.D. = 2.13 m/s).

In addition, there was a significant interaction between traffic volume and IDCTS on drivers’ mean speed (F = 3.434, *p* < 0.05). To further explore the interaction between traffic volume and IDCTS, pairwise comparisons were also conducted. The results in Table 5 revealed that the mean speed of vehicles at 100 m IDCTS for a traffic volume of 500 pcu/h was significantly higher than that for a traffic volume of 1000 pcu/h (M.D. = 1.277 *, *p* < 0.05) and a traffic volume of 1500 pcu/h (M.D. = 0.937, *p* < 0.05); for 150 m IDCTS, the mean speed of vehicles at 500 pcu/h was significantly higher than that at 1500 pcu/h (M.D. = 1.321, *p* < 0.001). The mean speed of vehicles at 500 pcu/h with IDCTS of 50 m was significantly lower than the mean speed of vehicles with IDCTS of 100 m (M.D. = −1.275, *p* < 0.05) and 150 m (M.D. = −1.447, *p* < 0.001).

**Figure 3:**
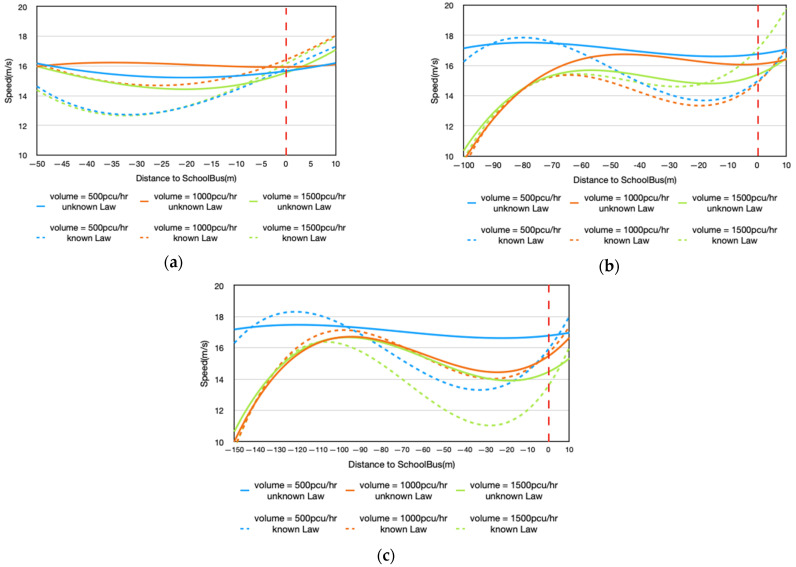
(**a**) Mean speed profiles under IDCTS = 50 m; (**b**) mean speed profiles under IDCTS = 100 m; (**c**) mean speed profiles under IDCTS = 150 m.

In order to thoroughly demonstrate the driving behavior of drivers while approaching the school bus, the speed profiles of all drivers were analyzed in detail. Linear interpolation and 1 m interval were used to deal with the speed data of different distances from the vehicle and the school bus at 1 m intervals. Figure 3 presents the speed profiles of all drivers under the influence of three factors, traffic volume and IDCTS, before and after learning about the school bus stopping regulations. The vertical dashed line, called the overtaking line, represents the overtaking position (the position when the relative distance between the front of the test vehicle and the rear of the school bus was 0 is the overtaking line). Figure 3 shows that the drivers’ speed fluctuation in the process of approaching the school bus before learning about the regulations is significantly smaller than that after learning about the regulations. It reflects the fact that after the driver knows the school bus stop regulations, there is a significant decrease in the speed value in the process of approaching the overtaking line and a significant increase in the speed value near the overtaking line (within 10 m to the left and right of the vertical dashed line). Besides, the speed increases greatly when the IDCTS is 100 m and the traffic flow is 1500 pcu/h (Figure 3b). In addition, after learning about the school bus stopping regulations, the driver’s speed decreases and the distance of deceleration increases gradually as the IDCTS increases (Figure 3b,c).

#### 3.2.2. Longitudinal Acceleration/Deceleration

The results of the MANOVA (see Table 4) showed that the effects of traffic law awareness (F = 33.532, *p* < 0.05), traffic volume (F = 9.360, *p* < 0.05), and IDCTS (F = 9.813, *p* < 0.05) on the driver’s maximum acceleration are significant. Traffic law awareness (F = 98.030, *p* < 0.001) and traffic volume (F = 3.743, *p* < 0.024) have a significant effect on the maximum deceleration of the driver.

The maximum acceleration (M = 3.541 m/s^2^, S.D. = 1.361 m/s^2^) and maximum deceleration (M = 4.930 m/s^2^, S.D. = 3.354 m/s^2^) of the driver after learning about the regulations were significantly greater than the maximum acceleration (M = 2.948 m/s^2^, S.D. = 0.985 m/s^2^) and maximum deceleration (M = 2.587 m/s^2^, S.D. = 2.567 m/s^2^) before learning about the regulations. The maximum acceleration (M = 2.991 m/s^2^, S.D. = 1.259 m/s^2^) and maximum deceleration (M = 2.587 m/s^2^, S.D. = 2.567 m/s^2^) at an IDCTS of 50 m were significantly smaller than those at an IDCTS of 100 m (M = 3.456 m/s^2^, S.D. = 1.119 m/s^2^) and 150 m (M = 3.152 m/s^2^, S.D. = 1.218 m/s^2^) cases. For different traffic volumes, the maximum acceleration and maximum deceleration showed an increasing trend when the traffic volume increased from 500 pcu/h to 1500 pcu/h.

The MANOVA results showed that there was a significant interaction between traffic law awareness, traffic volume, and IDCTS on drivers’ maximum acceleration (F = 1.107, *p* < 0.05), while there was no significant interaction between these factors on drivers’ maximum deceleration. The pairwise comparison result of maximum acceleration in Table 5 reveals significant group differences for multiple combinations of traffic volumes and IDCTS with knowledge of regulations. In particular, the maximum acceleration for a traffic volume of 500 pcu/h (M = 3.299 m/s^2^, S.D. = 0.184 m/s^2^) when the IDCTS is 100 m is significantly smaller than that for a traffic volume of 1000 pcu/h (M = 4.108 m/s^2^, S.D. = 0.184 m/s^2^). In addition, with an increase in traffic volume and IDCTS, the maximum acceleration of the driver also showed an increasing trend.

**Figure 4:**
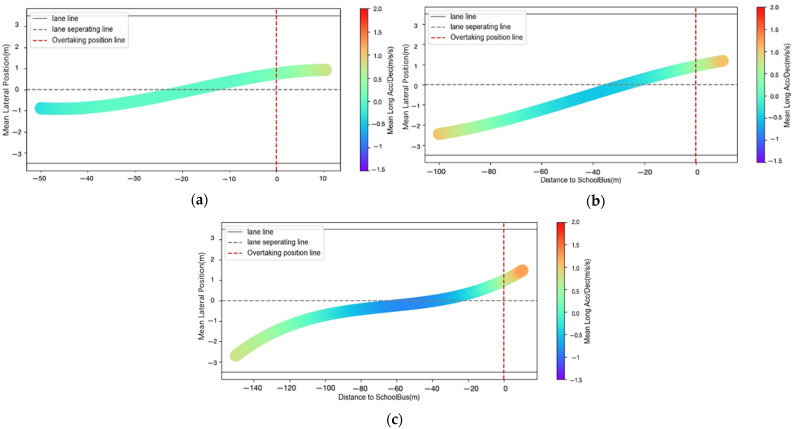
(**a**) Mean longitudinal Acc/Dec profiles when IDCTS = 50 m and volume = 500 pcu/h; (**b**) mean longitudinal Acc/Dec profiles when IDCTS = 100 m and volume = 1000 pcu/h; (**c**) mean longitudinal Acc/Dec profiles when IDCTS = 150 m and volume = 1500 pcu/h.

To better understand the acceleration/deceleration under different influencing factors, the acceleration/deceleration of the driver while approaching the school bus from different initial locations for each scenario are plotted in Figure 4. It indicates that, with an increase in traffic volume and IDCTS, the values of vehicle acceleration and deceleration, as well as the duration of acceleration and deceleration states, are significantly higher. Besides, the acceleration states are more obvious in two situations: before the vehicle’s lane changing when it is far away from the school bus, and when the vehicle is within or beyond the overtaking line. However, the deceleration state is mainly distributed in the lane change process.

#### 3.2.3. Lateral Acceleration

Among the 666 observations, 579 drivers chose to change the lane while driving behind the school bus. The MANOVA results (see Table 4) show that the drivers’ maximum lateral acceleration is significantly influenced by IDCTS (F = 10.992, *p* < 0.001). Specifically, there is a decreasing trend in lateral acceleration as IDCTS increases. By contrast, the knowledge of regulations (F = 0.216, *p* = 0.643) and traffic volume (F = 2.550, *p* = 0.079) does not have significant effects on lateral acceleration.

The results of MANOVA also show significant interactions between knowledge of regulations, traffic volume, and IDCTS (F = 10.992, *p* < 0.05). The results of pairwise comparisons are presented in Table 5. With knowledge of regulations, the lateral acceleration of the drivers at an IDCTS of 50 m with a traffic volume of 1000 pcu/h (M = 2.637 m/s^2^, S.D. = 0.011 m/s^2^) was significantly higher than that at a traffic volume of 500 pcu/h (M = 1.677 m/s^2^, S.D. = 0.012 m/s^2^) and 1500 pcu/h (M = 1.323 m/s^2^, S.D. = 0.012 m/s^2^). Besides, for 1000 pcu/h traffic volume, the lateral acceleration of the drivers at an IDCTS of 50 m (M = 2.637 m/s^2^, S.D. = 0.011 m/s^2^) and 100 m (M = 2.719 m/s^2^, S.D. = 0.011 m/s^2^) was significantly greater than that at IDCTS of 150 m (M = 1.299 m/s^2^, S.D. = 0.010 m/s^2^). 1.299 m/s^2^, S.D. = 0.010 m/s^2^).

**Figure 5:**
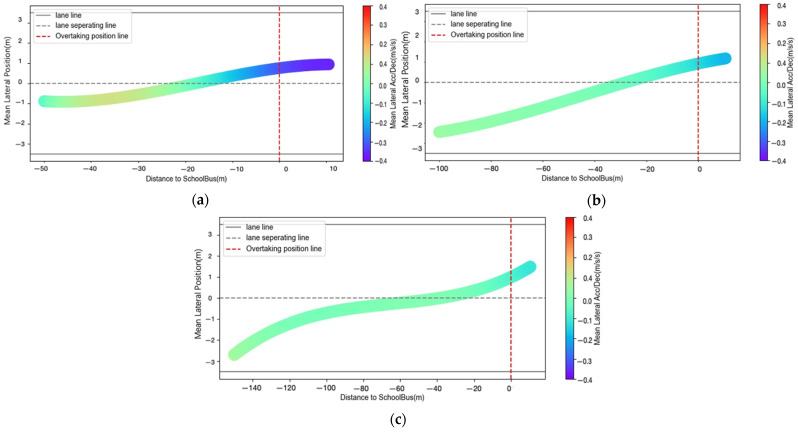
(**a**) Mean lateral Acc/Dec profiles when IDCTS = 50 m and volume = 500 pcu/h; (**b**) mean lateral Acc/Dec profiles when IDCTS = 100 m and volume = 1000 pcu/h; (**c**) mean lateral Acc/Dec profiles when IDCTS = 150 m and volume = 1500 pcu/h.

Figure 5 shows the driver’s average lateral acceleration during the approach to the school bus from different positions in the lane. It indicates that, with an increase in traffic volume and IDCTS, the variation of average lateral acceleration gradually decreases, while the duration of the lane change progress becomes longer. When the IDCTS and the traffic volume are small, the average lateral acceleration fluctuates relatively a lot (Figure 5a), which is related to the driver’s completion of lane change at a short distance and a high speed. However, in the case of sufficient lane change distance and speed reduction due to large traffic volume, the average lateral acceleration of the driver when changing lanes is smaller (Figure 5b,c).

#### 3.2.4. Percentage of Following Time

**Figure 6:**
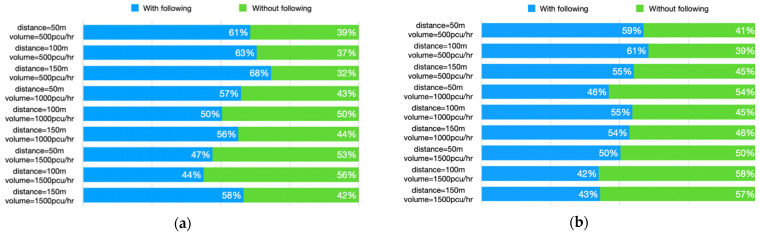
(**a**) Percentage of following time after learning about the school bus regulations; (**b**) percentage of following time if the school bus regulations are not known.

The MANOVA results (see Table 4) suggested that traffic law awareness (F = 5.070, *p* < 0.05) and traffic volume (F = 15.864, *p* < 0.001) have significant effects on the percentage of following time, and there is no interaction between the factors. The percentage of following time after learning about the regulations (M = 56.27%, S.D. = 24.75%) is significantly lower than that before learning about the regulations (M = 51.58%, S.D. = 23.23%). Besides, the percentage of following time tends to decrease as the traffic volume increases. The reason might be that the higher the traffic volume, the more the drivers tend to change lanes earlier after observing a school bus in front of them. The detail of the percentage of following time in each scenario is shown in Figure 6.

## 4. Discussion

The results of the study showed that there is a significant difference in drivers’ behavior before and after learning about the school bus stopping regulations. The driving behavior of the subjects in the experiment before they were aware of the regulations remained largely consistent with what we observed in reality, with the vast majority of drivers overtaking the school bus after it had stopped. It is clear from the test data that the average speed of the driver after learning about the regulations decreases significantly and the proportion of drivers who choose to stop after the school bus increases significantly. Simultaneously, the maximum deceleration of drivers increases significantly, which is coherent with their need to comply with law on stopping for a school bus. Changes in drivers’ behavioral characteristics reflect their increased awareness of the “privileges” of school buses, which are also considered to be one of the key factors influencing road safety [31]. Therefore, raising the level of driver awareness of school bus regulations is beneficial to improving the safety of school buses and surrounding vehicles.

Another finding is that after learning about the regulations, the percentage of both Acc state and Stop bus state increases. This indicates that although the publicity of regulations allows some drivers to comply with the law on stopping for a school bus, other drivers still choose to overtake the school bus to smoothly and quickly pass the school bus section. Evidence shows that driver motivation plays an essential role in choosing to drive in a certain way and can promote or prevent risky driving behavior [32,33,34]. Drivers are motivated to drive faster when there is time pressure to get to their destination on time, when they are more confident in their driving ability, and when they believe that they have more driving experience than others [35,36,37]. Thus, they increase their acceleration to overtake the school bus if they find that there is an opportunity to overtake before the school bus stops completely. This is the reason why the maximum acceleration of drivers is significantly higher after learning about the regulations. However, due to varying traffic volume and initial distance, as well as the fact that the school bus does not show the stop sign, it is difficult for drivers to judge whether the school bus has completely stopped. This might be the reason why the proportion of school buses in a stopped state while other vehicles overtake them has increased. It is worth noting that the increase in this behavior poses a safety hazard for school buses and surrounding vehicles, since urgent acceleration and deceleration are also part of unsafe driving behavior [20].

In addition, the results suggested that there are significant differences in the effects on the driver’s driving behavior under the combined scenarios of different traffic volumes and initial distances from the school bus. Specially, some scenarios have great crash risks. Since most school buses do not stop at a harbor and with no school bus stop sign on the side of the road, surrounding drivers driving behind the school bus do not know the specific stop location. According to the results, when the IDCTS was 100 m and 150 m and the traffic volume was 1000 pcu/h and 1500 pcu/h, the driving process had a higher frequency of sharp acceleration and deceleration. There might be two reasons: (1) some drivers chose to slow down but, when approaching the school bus, found that the school bus had not stopped, so they took the action of speeding up and overtaking; (2) some drivers directly chose to accelerate to overtake the school bus, but when approaching the school bus, they found that they could not overtake the school bus before stopping, so they began to take emergency braking actions. This, however, occurred in the context of relatively high traffic volumes and is consistent with the findings of Sevin Mohammadi et al. (2021), that the likelihood of acceleration of the subject vehicle is higher when there are more surrounding vehicles and when the difference in speed between the subject vehicle and the surrounding vehicles is small [38].

In summary, the stopping of school buses has a great influence on the behavior of vehicles following in the same lane. Although some drivers will drive more carefully after learning about the regulations, there are still some drivers who will find ways to pursue speed and time efficiency. There might be three reasons: (1) there is a mismatch between the increasing speed of school bus ownership in developing countries and the popularization of school bus-related laws and the intensity of law enforcement; (2) part of the time of school bus operation overlaps with the morning and evening peak hours, which exacerbate traffic congestion; (3) there is a “dilemma zone” for surrounding vehicles due to the lack of harbor and stop signs, which makes it difficult for them to judge whether it is safe to pass the school bus.

## 5. Conclusions

In this study, a driving simulator-based experiment was conducted in which a series of combined scenarios with different traffic volumes and IDCTS were designed using a driving simulator, and driver behavior data were collected. Accordingly, the driver’s performance during the school bus stop was investigated under various scenarios, such as whether they were aware of the school bus regulations, different traffic volumes, and the initial position of the school bus. The results show that after the promotion of laws and regulations, the speed of drivers dropped significantly, and the number of people who abide by the regulations increased, which indicates that legal education can, to a certain extent, increase the drivers’ awareness of school bus privileges. However, knowing the school bus stopping regulations also poses driving risks that cannot be ignored. The regulations will stimulate the driver to a certain extent to increase their rapid acceleration and deceleration behavior in pursuit of time efficiency. It was found that the driver’s speed fluctuations increase significantly when approaching the school bus, and the probability of accelerating and overtaking events increases, especially when the traffic volume is high and the initial position is far.

The findings of this study will help policy makers better understand the prevalence of and compliance with current school bus stopping regulations among drivers. Under the influence of various factors in developing countries (such as the current road environment, law enforcement, as well as the drivers’ traffic law awareness), school bus stopping regulations may not be known and complied with by all drivers within a short period of time after enactment. Therefore, there is a transitional period before the regulations are fully implemented (the regulations are not enforced). Our research can provide a basis for policy makers to assess the potential safety benefits and risks associated with the mandatory implementation of the regulations. As a result, it can help policy makers to develop management and education measures for the transition phase to reduce the driving safety risks associated with the mandatory implementation of the regulations.

Additionally, there are still many limitations that need to be addressed in the follow-up studies. First, sociological factors are not considered in this study. The influence of different drivers’ social attributes (age, education, family situation, distance/speed perception, etc.) on driving behaviors might be a useful perspective for finding strategies from education. Second, in this study, only drivers aged 21–46 years were recruited, and the age was concentrated between 23 and 30 years. Future analysis will be conducted on an expanded sample. Third, although we randomly select scenarios to avoid participants’ expectations of the experiment, it might still have a learning effect. In future experiments, events such as school buses decelerating but not stopping can be set to reduce the impact of learning effects. Furthermore, it is worth exploring whether the provision of school bus stop location information to vehicles around school buses through roadside stop signs and pre-stop warning signs for school buses, as well as the provision of school bus stop warning information to vehicles around school buses through Telematics early warning technology, could be an effective measure to improve the safety of school buses and surrounding vehicles in the future.

## Figures and Tables

**Table 1:** Description for Stop/Go decision, Acc/Dec state, and Stop/Go bus state.

Law	IDCTS (m)	Volume (pcu/h)	Stop/Go Decision				Acc/Dec State				Stop/Go Bus State				Total
			Stop		Go		Acc		Dec		Stop		Go		
			N	%	N	%	N	%	N	%	N	%	N	%	N
unknown	50	500	0	0.0	37	100.0	16	43.2	21	56.8	19	51.4	18	48.6	37
unknown	50	1000	1	2.7	36	97.3	20	55.6	16	44.4	18	50.0	18	50.0	37
unknown	50	1500	0	0.0	37	100.0	19	51.4	18	48.6	31	83.8	6	16.2	37
unknown	100	500	0	0.0	37	100.0	19	51.4	18	48.6	13	35.1	24	64.9	37
unknown	100	1000	0	0.0	37	100.0	14	37.8	23	62.2	17	45.9	20	54.1	37
unknown	100	1500	0	0.0	37	100.0	22	59.5	15	40.5	25	67.6	12	32.4	37
unknown	150	500	1	2.7	36	97.3	23	63.0	13	36.1	23	63.9	13	36.1	37
unknown	150	1000	0	0.0	37	100.0	21	56.8	16	43.2	17	45.9	20	54.1	37
unknown	150	1500	2	5.4	35	94.6	17	48.6	18	51.4	28	80.0	7	20.0	37
known	50	500	0	0.0	37	100.0	24	64.9	13	35.1	22	59.5	15	40.5	37
known	50	1000	4	10.8	33	89.2	22	66.7	11	33.3	25	75.8	8	24.2	37
known	50	1500	9	24.3	28	75.7	21	75.0	7	25.0	25	89.3	3	10.7	37
known	100	500	2	5.4	35	94.6	22	62.9	13	37.1	16	45.7	19	54.3	37
known	100	1000	3	8.1	34	91.9	24	70.6	10	29.4	18	52.9	16	47.1	37
known	100	1500	9	24.3	28	75.7	16	57.1	12	42.9	18	64.3	10	35.7	37
known	150	500	1	2.7	36	97.3	29	80.6	7	19.4	20	55.6	16	44.4	37
known	150	1000	2	5.4	35	94.6	23	65.7	12	34.3	19	54.3	16	45.7	37
known	150	1500	10	27.0	27	73.0	14	51.9	13	48.1	18	66.7	9	33.3	37

**Table 2:** Descriptive statistical results for dependent variables.

Factors			Mean Speed (m/s)		Max Long Acc (m/s^2^)		Max Long Dec (m/s^2^)		Max Lateral Acc (m/s^2^)		Percentage of Following Times (%)	
Traffic Flow	IDCTS	Law	Mean	S.D.	Mean	S.D.	Mean	S.D.	Mean	S.D.	Mean	S.D.
500 (pcu/h)	50 (m)	unknown	13.46 ^1^	1.97	2.62	1.43	2.00	1.97	2.04	1.70	0.11	0.17
		known	13.45 ^1^	2.18	3.29	0.78	4.73	3.63	1.61	2.06	0.24	0.31
	100 (m)	unknown	15.02 ^1^	1.81	2.74	1.45	2.49	2.49	1.60	1.48	0.63	0.23
		known	14.45 ^1^	1.57	3.30	0.92	4.79	3.39	1.61	1.50	0.61	0.23
	150 (m)	unknown	15.27 ^1^	2.36	2.71	1.47	1.94	1.86	1.78	1.81	0.68	0.22
		known	14.54 ^1^	2.50	3.70	0.50	5.02	3.45	0.83	0.85	0.55	0.23
1000 (pcu/h)	50 (m)	unknown	14.05 ^1^	2.09	2.52	1.45	2.14	2.49	1.45	1.07	0.57	0.25
		known	13.82 ^1^	2.13	3.14	1.35	4.25	3.32	2.65	2.61	0.46	0.23
	100 (m)	unknown	13.90 ^1^	1.81	2.79	1.50	2.30	2.44	2.18	2.49	0.50	0.25
		known	13.01 ^1^	2.16	4.11	0.31	5.16	3.24	2.82	3.34	0.55	0.25
	150 (m)	unknown	14.20 ^1^	2.76	2.85	1.42	2.86	2.73	1.84	1.87	0.56	0.25
		known	14.13 ^1^	2.65	3.02	1.26	4.95	3.34	1.31	1.18	0.54	0.22
1500 (pcu/h)	50 (m)	unknown	13.43 ^1^	1.91	2.66	1.30	2.63	2.54	2.43	2.39	0.47	0.27
		known	12.78 ^1^	2.83	3.71	0.58	4.79	3.57	1.22	1.25	0.50	0.24
	100 (m)	unknown	13.71 ^1^	2.15	4.00	0.46	3.32	3.20	2.02	1.68	0.44	0.23
		known	13.91 ^1^	2.16	3.79	0.56	5.31	3.41	1.39	1.31	0.42	0.19
	150 (m)	unknown	14.03 ^1^	2.33	3.63	0.62	3.62	2.86	1.11	0.81	0.58	0.21
		known	13.46 ^1^	2.91	3.01	1.36	5.36	3.04	0.96	0.85	0.43	0.22

^1^ Speed limit 70 km/h.

**Table 3:** Results of logistic regression model for Stop/Go decision, Acc/Dec state, and Stop/Go bus state.

Factors	B	S.E.	Wald	df	Sig.	Exp (B)	95% C.I. for EXP (B)	
							Lower	Upper
Stop/Go decision								
Law (reference level: unknown)								
known	−2.500	0.536	21.788	1	0.000	0.82	0.029	0.235
IDCTS (reference level: 50 m)			22.957	2	0.000			
100 m	−2.242	0.549	16.681	1	0.000	0.106	0.036	0.312
150 m	−1.277	0.389	10.762	1	0.001	0.279	0.130	0.598
Constant	−1.129	0.217	27.070	1	0.000	0.323		
Acc/Dec state								
Law (reference level: unknown)								
known	−0.609	0.166	13.480	1	0.000	0.544	0.393	0.753
Stop/Go bus state								
Gender (reference level: female)								
male	−0.484	0.175	7.678	1	0.006	0.616	0.437	0.868
Volume (reference level: 500 pcu/h)			11.592	2	0.003			
1000 (pcu/h)	−0.304	0.212	2.064	1	0.151	0.738	0.488	1.117
1500 (pcu/h)	0.405	0.206	3.869	1	0.049	1.499	1.001	2.243
IDCTS (reference level: 50 m)			28.247	2	0.000			
100 m	1.084	0.219	24.408	1	0.000	2.957	1.924	4.547
150 m	1.001	0.221	20.574	1	0.000	2.721	1.766	4.194
Constant	−0.995	0.220	20.550	1	0.000	0.370		

**Table 4:** Results of MANOVA for dependent variables.

Factors		df	F	Sig.	*η* ^2^
**Mean Speed (m/s)**	Law	1	5.887	0.016	0.009
	Volume	2	8.179	0.000	0.025
	IDCTS	2	5.868	0.003	0.018
	Law * Volume	2	0.011	0.990	0.000
	Law * IDCTS	2	0.192	0.825	0.001
	Volume * IDCTS	4	3.434	0.009	0.021
	Law * Volume * IDCTS	4	1.107	0.352	0.007
**Max Long Acc (m/s^2^)**	Law	1	33.532	0.000	0.049
	Volume	2	9.360	0.000	0.028
	IDCTS	2	9.813	0.000	0.029
	Law * Volume	2	6.128	0.002	0.019
	Law * IDCTS	2	4.035	0.018	0.012
	Volume * IDCTS	4	2.993	0.018	0.018
	Law * Volume * IDCTS	4	6.394	0.000	0.038
**Max Long Dec (m/s^2^)**	Law	1	98.030	0.000	0.131
	Volume	2	3.743	0.024	0.011
	Distance	2	2.738	0.065	0.008
	Law * Volume	2	0.717	0.489	0.002
	Law * IDCTS	2	0.039	0.962	0.000
	Volume * IDCTS	4	0.459	0.766	0.003
	Law * Volume * IDCTS	4	0.402	0.807	0.002
**Max Lateral Acc (m/s^2^)**	Law	1	0.216	0.643	0.000
	Volume	2	2.550	0.079	0.009
	IDCTS	2	10.992	0.000	0.038
	Law * Volume	2	6.878	0.001	0.024
	Law * IDCTS	2	2.157	0.117	0.008
	Volume * IDCTS	4	0.421	0.793	0.003
	Law * Volume * IDCTS	4	3.249	0.012	0.023
**Percentage of Following Times (%)**	law	1	5.070	0.025	0.009
	Volume	2	15.864	0.000	0.054
	IDCTS	2	1.010	0.365	0.004
	Law * Volume	2	0.150	0.861	0.001
	Law * IDCTS	2	2.480	0.085	0.009
	Volume * IDCTS	4	0.610	0.655	0.004
	Law * Volume * IDCTS	4	1.539	0.190	0.011

* denotes the interaction effect between two variables.

**Table 5:** Result of pairwise comparisons.

Factors	Mean Difference	Std. Error	Sig.
Mean Speed (m/s)			
IDCTS 100 m (Volume 500 pcu/h) and IDCTS 100 m (Volume 1000 pcu/h)	1.277 *	0.373	0.002
IDCTS 150 m (Volume 500 pcu/h) and IDCTS 100 m (Volume 1500 pcu/h)	1.321 *	0.373	0.001
Volume 500 pcu/h (IDCTS 50 m) and Volume 500 pcu/h (IDCTS 100 m)	−1.275 *	0.373	0.002
Volume 500 pcu/h (IDCTS 50 m) and Volume 500 pcu/h (IDCTS 150 m)	−1.447 *	0.373	0.000
Max Long Acc (m/s^2^)			
Know law and IDCTS 100 m (Volume 500 pcu/h) and IDCTS 100 m (Volume 1000 pcu/h)	−0.809 *	0.261	0.006
Know law and Volume 1000 pcu/h (IDCTS 50 m) and Volume 1000 pcu/h (IDCTS 100 m)	−0.967 *	0.216	0.001
Know law and Volume 1000 pcu/h (IDCTS 100 m) and Volume 1000 pcu/h (IDCTS 150 m)	1.090 *	0.216	0.000
Know law and Volume 1500 pcu/h (IDCTS 100 m) and Volume 1500 pcu/h (IDCTS 150 m)	0.781 *	0.261	0.009
Max Lateral Acc (m/s^2^)			
Know law and IDCTS 50 m (Volume 500 pcu/h) and IDCTS 50 m (Volume 1000 pcu/h)	−0.327	0.150	0.090
Know law and IDCTS 50 m (Volume 1000 pcu/h) and IDCTS 50 m (Volume 1500 pcu/h)	0.474 *	0.152	0.006
Know law and Volume 1000 pcu/h (IDCTS 50 m) and Volume 1000 pcu/h (IDCTS 150 m)	0.483 *	0.145	0.003
Know law and Volume 1000 pcu/h (IDCTS 100 m) and Volume 1000 pcu/h (IDCTS 150 m)	−0.509 *	0.148	0.002

* denotes there is significant difference.

## Data Availability

The data presented in this study are available on request from the corresponding author.
